# Association between COVID-19 vaccination and critical outcomes among older adults with dementia: a comparative cohort study

**DOI:** 10.3389/fpubh.2023.1281266

**Published:** 2023-10-02

**Authors:** Zorian Radomyslsky, Sara Kivity, Shira Lidar, Netta Bentur, Liat Korn, Rachel Nissanholtz-Gannot, Shelley Sternberg, Inbal Halevi Hochwald, Orna Reges, Yaniv Alon, Mor Saban

**Affiliations:** ^1^Department of Health System Management, Ariel University, Ariel, Israel; ^2^Department of Occupational Therapy, School of Health Sciences, Faculty of Medicine, Tel Aviv University, Tel Aviv, Israel; ^3^Department of Health System Management, Ariel University, Ariel, Israel; ^4^Mayers-JDC-Brookdale Institute, Jerusalem, Israel; ^5^School of Nursing, Max Stern Yezreel Valley College, Emek Yezreel, Israel; ^6^Nursing Department, School of Health Sciences, Faculty of Medicine, Tel Aviv University, Tel Aviv, Israel

**Keywords:** dementia, COVID-19, mild cognitive impairment, vaccination, psychiatric disorder diagnosis

## Abstract

**Background:**

As COVID-19 vaccines became available, understanding their potential benefits in vulnerable populations has gained significance. This study explored the advantages of COVID-19 vaccination in individuals with cognitive disorders by analyzing health-related variables and outcomes.

**Methods:**

A prospective cohort study analyzed electronic medical records of 25,733 older adults with cognitive disorders and 65,544 older adults without cognitive disorders from March 2020 to February 2022. COVID-19 vaccination status was the primary exposure variable, categorized as fully vaccinated or unvaccinated. The primary outcomes measured were all-cause mortality and hospitalization rates within 14 and 400 days post-vaccination. Data on vaccination status, demographics, comorbidities, testing history, and clinical outcomes were collected from electronic health records. The study was ethically approved by the relevant medical facility’s Institutional Review Board (0075-22-MHS).

**Results:**

Vaccinated individuals had significantly lower mortality rates in both groups. In the research group, the mortality rate was 52% (*n* = 1852) for unvaccinated individuals and 7% (*n* = 1,241) for vaccinated individuals (*p* < 0.001). Similarly, in the control group, the mortality rate was 13.58% (*n* = 1,508) for unvaccinated individuals and 1.85% (*n* = 936) for vaccinated individuals (*p* < 0.001), despite higher COVID-19 positivity rates. In the research group, 30.26% (*n* = 1,072) of unvaccinated individuals tested positive for COVID-19, compared to 37.16% (*n* = 6,492) of vaccinated individuals (*p* < 0.001). In the control group, 17.31% (*n* = 1922) of unvaccinated individuals were COVID-19 positive, while 37.25% (*n* = 18,873) of vaccinated individuals tested positive (*p* < 0.001). Vaccination also showed potential benefits in mental health support. The usage of antipsychotic drugs was lower in vaccinated individuals (28.43%, *n* = 4,967) compared to unvaccinated individuals (37.48%, *n* = 1,328; 95% CI [0.92–1.28], *p* < 0.001). Moreover, vaccinated individuals had lower antipsychotic drug prescription rates (23.88%, *n* = 4,171) compared to unvaccinated individuals (27.83%, *n* = 968; 95% CI [−1.02 to −0.63], *p* < 0.001). Vaccination appeared to have a positive impact on managing conditions like diabetes, with 38.63% (*n* = 6,748) of vaccinated individuals having diabetes compared to 41.55% (*n* = 1,472) of unvaccinated individuals (95% CI [0.24, 0.48], *p* < 0.001).

**Discussion:**

The findings highlight the importance of vaccination in safeguarding vulnerable populations during the pandemic and call for further research to optimize healthcare strategies for individuals with cognitive disorders.

## Background

The emergence of the novel coronavirus SARS-CoV-2 led to the global COVID-19 pandemic that profoundly impacted public health systems and caused significant morbidity and mortality worldwide. As the virus rapidly spread across borders, healthcare providers and vulnerable populations including the older adult and those with chronic conditions faced tremendous challenges. Individuals with dementia have been disproportionately affected by COVID-19, experiencing accelerated cognitive decline, exacerbated behavioral and psychological symptoms, and increased care needs (1–3). Dementia encompasses progressive neurological disorders that impair higher cognitive functions including memory, language, and thinking. These cognitive impairments are accompanied by a decline in the performance of daily activities and a deterioration in social functioning (4). Globally, the prevalence of dementia is estimated to be around 50 million individuals, with approximately 60% of cases found in low- and middle-income countries (5). However, the global prevalence is predicted to increase to over 150 million cases by 2050 (6).

During the pandemic, individuals with dementia were shown to be at higher risk for adverse outcomes from COVID-19 infection, including increased mortality, morbidity, and acceleration of cognitive and functional decline (7, 8). For example, a recent meta-analysis showed that the mortality rate of individuals with dementia after being infected with COVID-19 was higher than that of individuals with no dementia (OR: 5.17 [95% CI: 2.31–11.59]) (9).

The literature reports an increase in behavioral issues, cognitive decline, and the use of antipsychotic medications, along with more frequent hospitalizations during this time (10, 11). This decline arose due to the lack of formal caregivers, unemployment among individuals with dementia, and substantial physical isolation, especially during lockdowns (2, 12). Individuals with dementia self-reported experiencing a lack of engagement, disrupted routines, and reduced physical activity during lockdowns. These factors were found to consequently contribute to documented cases of depression in multiple studies (11).

Israel’s COVID-19 vaccination program and policies have played a significant role in mitigating the impact of the pandemic. Israel implemented an aggressive and successful vaccination campaign, achieving high vaccination rates in its population. The country prioritized vaccinating older adults and individuals with underlying health conditions, including those with dementia. These efforts aimed to protect vulnerable populations and reduce the severity of COVID-19 outcomes (13–15).

The introduction of COVID-19 vaccinations had some effect on these outcomes. A handful of studies demonstrated the benefits of COVID-19 vaccination for reducing infection, hospitalization, and death in the general population, older adult, and specifically among those with dementia (16, 17). However, the precise influence of COVID-19 vaccination on severe outcomes among older adults with dementia remains unclear (18). A recent study published in 2022 (17) revealed that among older adults with dementia who were fully vaccinated, the overall risk of COVID-19 breakthrough infections ranged from 8.6 to 12.4% which was higher than that of older adults without dementia. There is also limited data available on the outcomes of older adults with dementia who received the COVID-19 vaccination compared to those who did not, across all phases of the pandemic (17).

The main objective of this study was to compare the long-term mortality rate between older adults with dementia and those without dementia, differentiated by whether they received the COVID-19 vaccination or not. We also aimed to compare the hospitalization rates between these two population groups.

## Methods

We conducted a prospective cohort study of older adults living in the community and insured under Maccabi Healthcare Services (MHS), one of the largest health management organizations in Israel. In MHS, there are over 300,000 insured individuals aged 65 and above. In 2019, MHS established the Cognitive Disorders Registry, which allows for the tracking of patients with cognitive decline in various clinical, therapeutic, managerial, and supportive arenas. Further detailed descriptions of the criteria for entering the registry can be found in Appendix 1. Approximately 25,000 insured individuals are included in this database. Some are defined as being in the pre-dementia stage with mild cognitive impairment (MCI), while others suffer from rare dementias.

In our study, we used the Clinical Dementia Rating (CDR) scale to assess the severity of cognitive impairment in our participants. The CDR is a widely used tool for assessing the severity of dementia and is based on a semi-structured interview with both the patient and an informant, such as a family member or caregiver (19).

The CDR scale ranges from 0 to 3, with 0 indicating no cognitive impairment, 0.5 indicating very mild cognitive impairment, 1 indicating mild dementia, 2 indicating moderate dementia, and 3 indicating severe dementia. In our study, we used a CDR score of 0.5 or greater to identify participants with dementia. To differentiate mild cognitive impairment (MCI) from dementia, we used the criteria established by Petersen et al. (20), which define MCI as a cognitive decline that is greater than expected for a person’s age and education level, but does not significantly interfere with their daily activities or meet the criteria for dementia. In our study, participants with a CDR score of 0.5 were classified as having dementia, while those with a CDR score of less than 0.5 were classified as having MCI or no cognitive impairment.

Electronic medical records spanning from March 01, 2020, to February 28, 2022, were examined for individuals who had continuous membership in MHS during the 2 years prior to the study date. The analysis focused on MHS insured individuals who were registered with cognitive decline in the Cognitive Disorders Registry who had either received or not received a COVID-19 vaccine (Pfizer/BioNTech or Moderna). The individuals enrolled in the registry were compared to a matched group (age, gender, and socioeconomic status) enrolled in MHS’s general national database who had either received the same COVID-19 vaccine or had not received one but did not experience cognitive decline.

### Study outcomes

The primary endpoint focused on the mortality rate within two timeframes: the first 14 days after the second vaccination for vaccinated individuals, corresponding to the expected peak of vaccine efficacy, vs. approximately 400 days for unvaccinated individuals, ending at the study closure on February 28, 2022.

The timeframe of 14 days post-second dose was chosen as the primary endpoint to assess peak vaccine efficacy. While the Pfizer and Moderna clinical trials reported slightly different windows for maximum efficacy (7 and 14 days, respectively), a conservative 14-day endpoint was selected for this study. This allows the analysis to sufficiently capture the protective effects across both mRNA vaccines, in line with public health guidance considering someone fully vaccinated at this threshold (21, 22).

Examining the immediate post-vaccination period allows a precise evaluation of the vaccine’s prompt impact at the anticipated height of immune response. The extended 400-day duration enables a comprehensive assessment of prolonged mortality trends in unvaccinated individuals over the same interval as vaccinated individuals. This elongated observation window accommodates variability in COVID-19 exposure and severity over time, accounting for potential deferred vaccination effects or shifting disease dynamics. The rationale for these timeframes also applies to our secondary outcome of hospitalization rates. Hospitalizations within 14 days of the second vaccine dose were analyzed for vaccinated individuals. Hospitalization cases for both groups were classified as either zero for no hospitalization or 1+ for one or more hospitalizations. Moreover, hospitalizations beyond the first 14 days were evaluated in both groups, particularly severe hospitalizations lasting 49+ days in unvaccinated and vaccinated individuals during the post-vaccination period.

### Data collection

Data collection for each patient encompassed a wide range of variables. The primary independent variable was vaccination status that was categorized based on whether the patient received two doses or more. Consequently, patients who received only one dose or none were considered as not vaccinated (mRNA vaccines or viral vector vaccines). Other independent variables collected were (1) information regarding sociodemographic characteristics, such as age, gender, district, and socioeconomic status; (2) COVID-19 characteristics, including the disease itself and the number of vaccine dosages received; and (3) clinical characteristics, including testing positive for COVID-19, transitioning from MCI to dementia, taking antipsychotics drugs, being prescribed antipsychotics drugs, taking antidepressants, being prescribed antidepressants, having a diagnosis of depression, receiving home treatments, diagnosed with bone fractures, and having blood pressure, chronic obstructive pulmonary disease (COPD), diabetes mellitus (DM), immunosuppression, or obesity.

### Statistical analysis

Descriptive statistics, including means and standard deviations for continuous variables, and percentages for categorical variables, were utilized to characterize the sociodemographic characteristics, COVID-19 characteristics, and clinical characteristics of the participants. To assess the statistical differences between groups, *t*-tests were employed for continuous variables, while chi-squared tests were used for categorical variables, as appropriate.

To reduce potential confounding factors arising from differences in patient characteristics between the vaccinated and unvaccinated groups within the cognitive disorders research group, we employed inverse probability of treatment weighting (IPTW). Standardized differences were utilized to evaluate the balance of covariates following IPTW implementation. Logistic regression was then performed, utilizing full vaccination status as the independent variable in the IPTW model. In addition, a Cox proportional hazards model with IPTW was conducted to analyze the occurrence of outcome events.

A survival analysis was performed for the older adults who passed away. The primary outcome variable was time to death, constructed as the time between the date of being vaccinated and death (failure) with censoring on February 28, 2022, for individuals who were alive by the end of the study period. We also included two secondary outcomes: (i) time from being vaccinated to hospital admission, and (ii) in-hospital length of stay. The Kaplan–Meier method was used to plot survival curves. These graphs served to test the proportional hazard assumption.

The level of significance for all statistical analyses was 5%. The data analysis was performed using Python (version 3.0).

### Ethical consideration

The study protocol was approved by the Institutional Human Subjects Ethics Committee (0075-22-MHS) of the relevant medical facility. Written informed consent was waived by the Institutional Review Board. All performed procedures followed the ethical standards of both the institutional and national research committees and these complied with national ethical standards.

## Results

**Figure 1:**
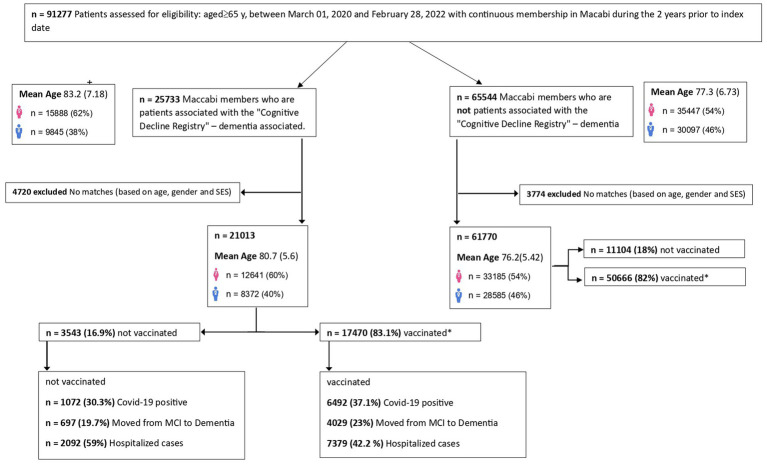
Flow chart depicting the population distribution of vaccinated vs. non-vaccinated groups. Vaccinated refers to individuals who received two vaccine shots against COVID-19.

Figure 1 presents the study population. A total of 25,733 older adults with cognitive disorders comprised the research group, of which 21,013 were investigated; 4,720 were excluded due to no matches (based on age, gender, and socioeconomic status). Among them, 3,543)16.9%(individuals did not receive the COVID-19 vaccination, while 17,470 (83.1%) individuals did receive the vaccination. The control group comprised 65,544 older adults without cognitive disorders, 3,774 were excluded due to no matches, resulting in a final total of 59,989 individuals. Among them, 10,768 (18%) individuals did not receive the COVID-19 vaccination, while 49,221 (82%) did receive the vaccination.

Within the research group, it was observed that 30.3% of the unvaccinated older adults tested positive for COVID-19. Moreover, 19.7% of the unvaccinated older adults experienced a progression from MCI to dementia, and 59% of them required hospitalization. In contrast, within the vaccinated research group, 37.1% of the older adults tested positive for COVID-19 (66.5% before receiving the vaccination and 33.5% after receiving the vaccination). Among the cohort of individuals diagnosed with dementia, 23% exhibited a progression from MCI to dementia, and 42.2% necessitated hospitalization, as depicted in Figure 1. Within the subgroup of vaccinated dementia-diagnosed individuals, 42% required hospitalization, with 3% experiencing prolonged hospitalization. In contrast, among the unvaccinated dementia-diagnosed individuals, 57% required hospitalization, with nearly 8% undergoing prolonged hospitalization. These observations underscore the potential benefits of vaccination in reducing the severity of hospitalization needs among individuals diagnosed with dementia during the study period.

Table 1 shows that in the research group, a substantial proportion of individuals in the research group opted for vaccination, with 83.1% vaccinated, while 16.9% remained unvaccinated. Similarly, in the control group, 82.0% of individuals chose vaccination, leaving 18.0% unvaccinated. Notably, the analysis revealed that the gender distribution between the vaccinated and unvaccinated groups did not show any statistically significant difference, implying that vaccination status was not influenced by gender. Furthermore, the research group demonstrated a higher mean age of 82.1 years for unvaccinated individuals and 80.6 years for the vaccinated group (*p* < 0.001). This was in contrast to the control group, where the unvaccinated individuals had a mean age of 77.9 years, and the vaccinated group had a mean age of 76.0 years (*p* < 0.001). 30.26% of unvaccinated individuals tested positive for COVID-19, whereas 37.16% of vaccinated individuals were COVID-19 positive (*p* < 0.001) in the research group. In the control group, 17.31% of unvaccinated individuals tested positive, while the number was 37.25% for vaccinated individuals (*p* < 0.001). Despite the higher rates of COVID-19 positivity and progression from MCI to dementia in the vaccinated research group, the mortality rates were significantly less in the vaccinated groups. In the control group, unvaccinated individuals had a mortality rate of 13.58% compared to only 1.85% of vaccinated individuals (*p* < 0.001). The difference was even more pronounced in the research group with 52.27% of unvaccinated individuals compared to 7.10% of vaccinated individuals dying (*p* < 0.001).

**Table 1:** COVID-19 vaccination and health outcomes in the research group with cognitive disorders compared to the control group.

	Research group					Control group				
	Unvaccinated		Vaccinated			Unvaccinated		Vaccinated		
	*n*	%	*n*	%	*p* value	*n*	%	*n*	%	*p* value
Sample size	3,543	16.86%	17,470	83.14%		11,104	17.98%	50,666	82.02%	
Male	1,407	39.70%	6,965	39.87%	0.86	5,091	45.85%	23,494	46.37%	0.37
Female	2,136	60.30%	10,505	60.13%	0.86	6,013	54.15%	27,172	53.63%	0.37
Age Mean (SD)	82.1 (5.46)		80.62 (5.58)		*p* < 0.001	77.88 (6.17)		75.98 (5.18)		*p* < 0.001
Mortality	1852	52.27%	1,241	7.10%	*p* < 0.001	1,508	13.58%	936	1.85%	*p* < 0.001
Positive for COVID-19	1,072	30.26%	6,492	37.16%	*p* < 0.001	1922	17.31%	18,873	37.25%	*p* < 0.001
Moved from MCI to dementia	697	19.67%	4,029	23.06%	*p* < 0.001					
Purchased antipsychiatry drugs	1,328	37.48%	4,967	28.43%	*p* < 0.001	506	4.56%	2,158	4.26%	0.17
Prescribed antipsychotics drugs	986	27.83%	4,171	23.88%	*p* < 0.001	305	2.75%	1866	3.68%	*p* < 0.001
Purchased antidepressants	1,553	43.83%	9,599	54.95%	*p* < 0.001	1,221	11.00%	12,586	24.84%	*p* < 0.001
Prescribed anti-depressants	1,354	38.22%	9,040	51.75%	*p* < 0.001	1,146	10.32%	12,704	25.07%	*p* < 0.001
Depression diagnosis	1,054	29.75%	5,750	32.91%	*p* < 0.01	1,250	11.26%	5,185	10.23%	*p* < 0.01
Home treatments	912	25.74%	1892	10.83%	*p* < 0.001	468	4.21%	630	1.24%	*p* < 0.001
Bone fractures	257	7.25%	2014	11.53%	*p* < 0.001	364	3.28%	4,049	7.99%	*p* < 0.001
Registered blood pressure	2,936	82.87%	13,743	78.67%	*p* < 0.05	7,714	69.47%	35,135	69.35%	0.89
Registered COPD	329	9.29%	1786	10.22%	0.11	1,154	10.39%	4,368	8.62%	*p* < 0.001
Registered diabetes	1,472	41.55%	6,748	38.63%	*p* < 0.05	3,847	34.65%	15,987	31.55%	*p* < 0.001
Registered Immunosuppression	893	25.20%	3,626	20.76%	*p* < 0.001	3,082	27.76%	8,470	16.72%	*p* < 0.001
Registered obesity	549	15.50%	4,617	26.43%	*p* < 0.001	1,551	13.97%	15,090	29.78%	*p* < 0.001

MCI, mild cognitive impairment.

The Cox ratio analysis of the research group affirmed the findings presented in Table 1. It revealed that the vaccinated group exhibited a substantially lower risk of mortality, with an adjusted hazard ratio (aHR) of −2.99 (95% CI: −2.86 to −3.12, *p* < 0.001). These results emphasize that vaccination provided significant protection against severe outcomes in this vulnerable population (Table 2). Additionally, the Cox ratio analysis illustrated notable associations with medication usage. The vaccinated group displayed a higher usage of antipsychotics drugs, with an aHR of 1.1 (95% CI: 0.92–1.28, *p* < 0.001), compared to the unvaccinated group, and lower prescription rates for antipsychotics drugs, with an aHR of −0.82 (95% CI: −1.02 to −0.63, *p* < 0.001), indicating potential differences in mental health management. Yet, the vaccinated group exhibited a higher usage of antidepressants, with an aHR of 0.54 (95% CI: 0.35–0.74, *p* < 0.001).

**Table 2:** Cox ratio analysis reveals the influence of COVID-19 vaccination on health outcomes and medication usage in the research group with cognitive disorders.

		Not vaccinated		Vaccinated		Adjusted hazard ratio			
		*n*	%	*n*	%	coef	coef lower 95%	coef upper 95%	*p* value
		3,543	16.86%	17,470	83.14%	−2.99	−3.12	−2.86	*p* < 0.001
Mortality	No	1,691	47.73%	16,229	92.90%				
	Yes	1852	52.27%	1,241	7.10%				
Positive to COVID-19	No	2,471	69.74%	10,978	62.84%	−1.1	−1.25	−0.96	*p* < 0.001
	Yes	1,072	30.26%	6,492	37.16%				
Moved from MCI to dementia	No	2,846	80.33%	13,441	76.94%	0.17	0.04	0.3	*p* < 0.01
	Yes	697	19.67%	4,029	23.06%				
Purchased antipsychiatry drugs	No	2,215	62.52%	12,503	71.57%	1.1	0.92	1.28	*p* < 0.001
	Yes	1,328	37.48%	4,967	28.43%				
Prescribed antipsychotics drugs	No	2,557	72.17%	13,299	76.12%	−0.82	−1.02	−0.63	*p* < 0.001
	Yes	986	27.83%	4,171	23.88%				
Purchased antidepressants	No	1990	56.17%	7,871	45.05%	0.54	0.35	0.74	*p* < 0.001
	Yes	1,553	43.83%	9,599	54.95%				
Prescribed antidepressants	No	2,189	61.78%	8,430	48.25%	−0.42	−0.62	−0.22	*p* < 0.001
	Yes	1,354	38.22%	9,040	51.75%				
Depression diagnosis	No	2,489	70.25%	11,720	67.09%	−0.25	−0.38	−0.12	*p* < 0.001
	Yes	1,054	29.75%	5,750	32.91%				
Home treatments	No	2,631	74.26%	15,578	89.17%	0.25	0.11	0.39	*p* < 0.001
	Yes	912	25.74%	1892	10.83%				
Bone fractures	No	3,286	92.75%	15,456	88.47%	−0.14	−3.22	2.94	0.46
	Yes	257	7.25%	2014	11.53%				
Hip fracture	No	3,286	92.75%	15,456	88.47%	−0.14	−3.22	2.94	0.46
	Yes	257	7.25%	2014	11.53%				
Registered blood pressure	No	607	17.13%	3,727	21.33%	0.07	−0.07	0.22	0.17
	Yes	2,936	82.87%	13,743	78.67%				
Registered COPD	No	3,214	90.71%	15,684	89.78%	0.19	0.01	0.37	*p* < 0.05
	Yes	329	9.29%	1786	10.22%				
Registered diabetes	No	2071	58.45%	10,722	61.37%	0.36	0.24	0.48	*p* < 0.001
	Yes	1,472	41.55%	6,748	38.63%				
Registered immunosuppression	No	2,650	74.80%	13,844	79.24%	0.52	0.4	0.64	*p* < 0.001
	Yes	893	25.20%	3,626	20.76%				
Registered obesity	No	2,994	84.50%	12,853	73.57%	−5.14	−6.41	−3.87	*p* < 0.001
	Yes	549	15.50%	4,617	26.43%				

The Cox proportional hazard model was constructed for the outcome events of the two study groups considering a previous history of outcome events, having tested positive to COVID-19, using antipsychotics drugs, using antidepressants, having depression diagnosis, bone fractures, and different registered medical conditions as covariates, weighted by the inverse probability of full vaccination.

**Figure 2:**
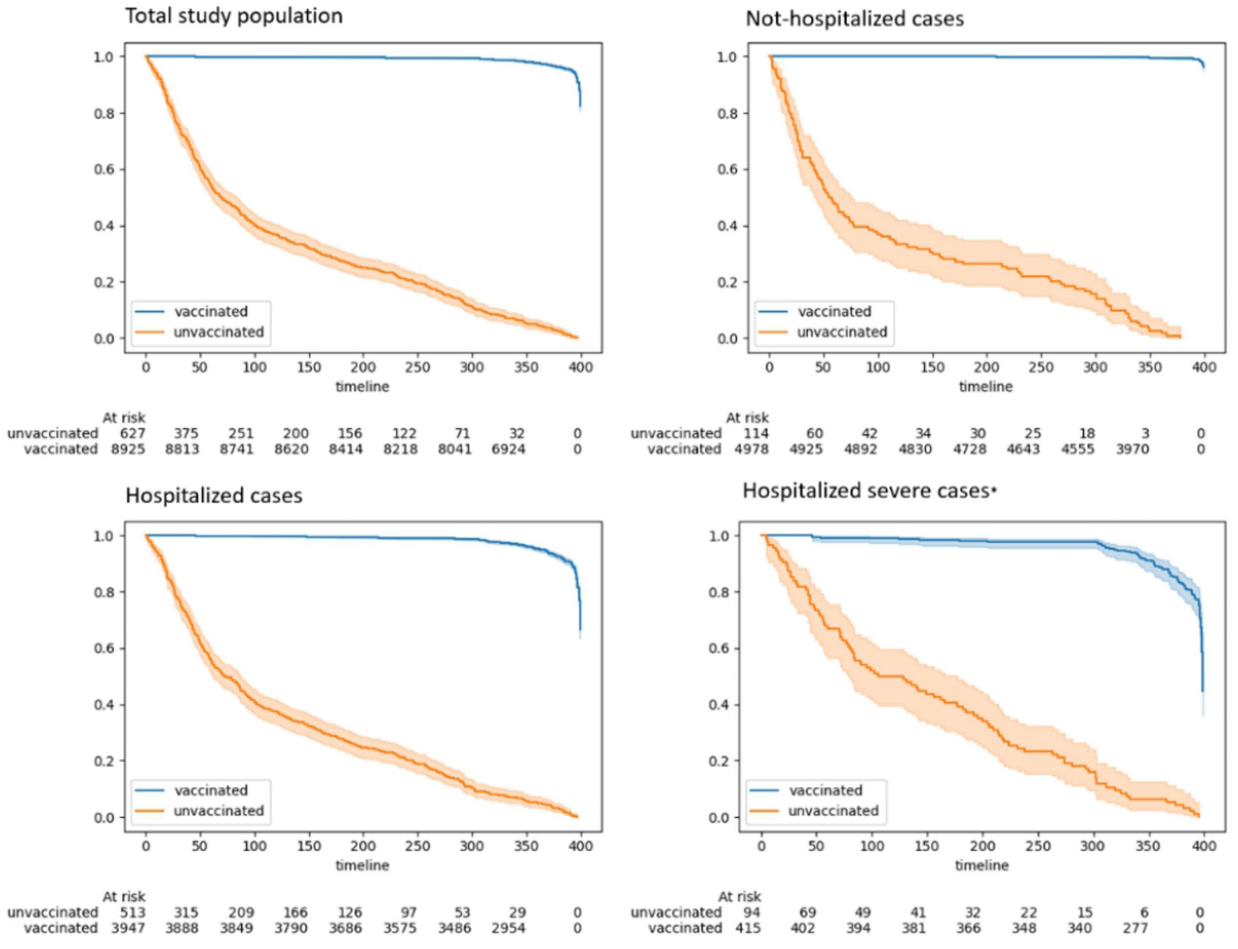
Kaplan–Meier analysis: the impact of vaccination on hospitalization severities in patients with cognitive disorders during the COVID-19 pandemic.

The Kaplan–Meier analysis examined the impact of COVID-19 vaccination on the research group during the pandemic, specifically focusing on the severity of their hospitalization (Figure 2). The analysis revealed that the survival curve for adults with cognitive disorders who were vaccinated was significantly higher compared to the survival curve for those who were unvaccinated. This indicates that vaccinated adults with cognitive disorders had a higher likelihood of surviving COVID-19 compared to their unvaccinated counterparts.

**Figure 3:**
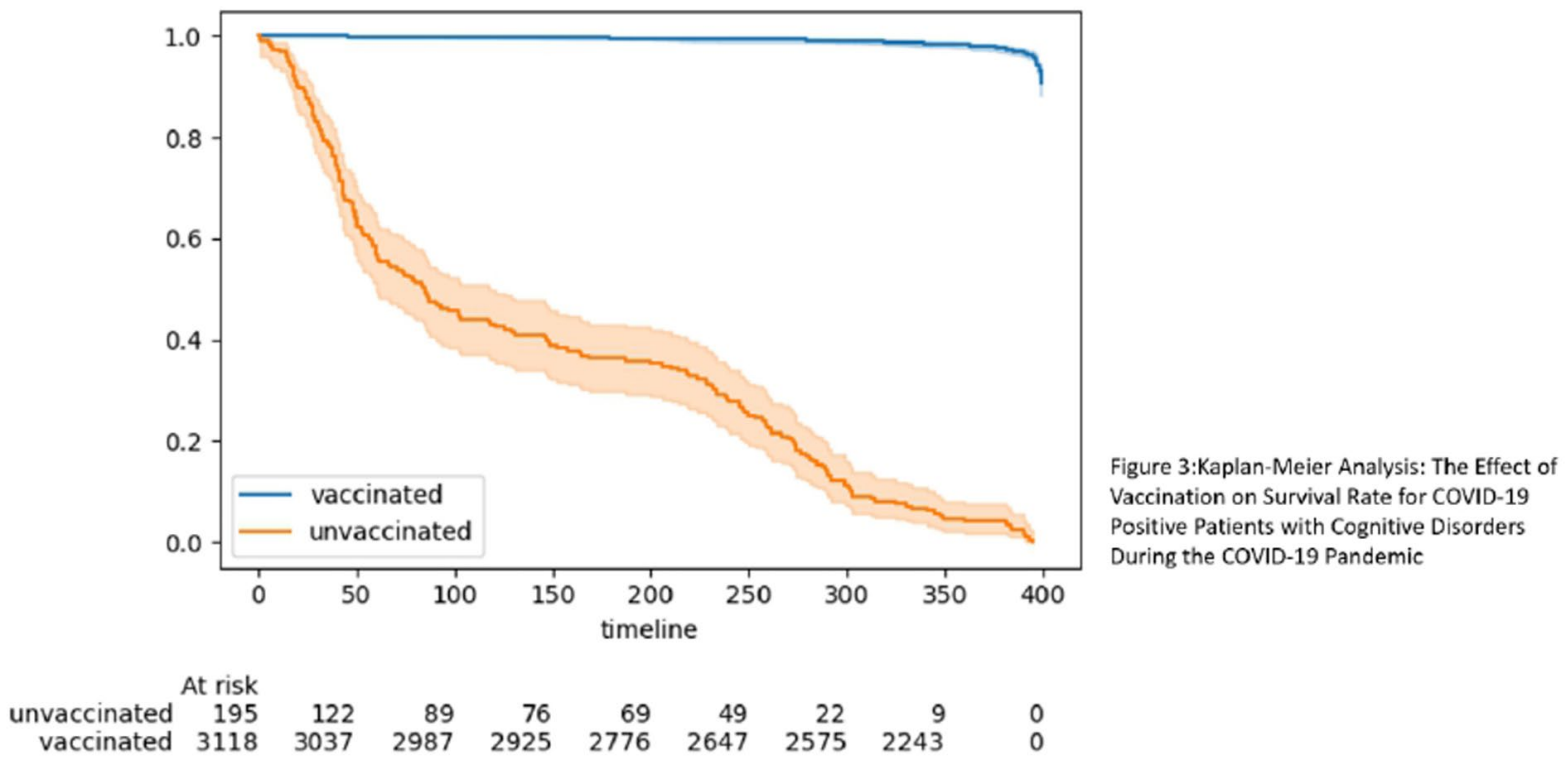
Kaplan–Meier analysis: the effect of vaccination on survival rate for COVID-19 positive patients with cognitive disorders during the COVID-19 pandemic.

The difference in survival rates between vaccinated and unvaccinated older adults is more crucial among those who experienced severe illness. In pursuit of comprehending the vaccination’s impact on survival rates, we performed a Kaplan–Meier analysis on adults with cognitive disorders who tested positive for COVID-19 (Figure 3). The findings illustrated that over time, vaccinated individuals, even those who tested positive for COVID-19, exhibited a higher likelihood of survival.

**Figure 4:**
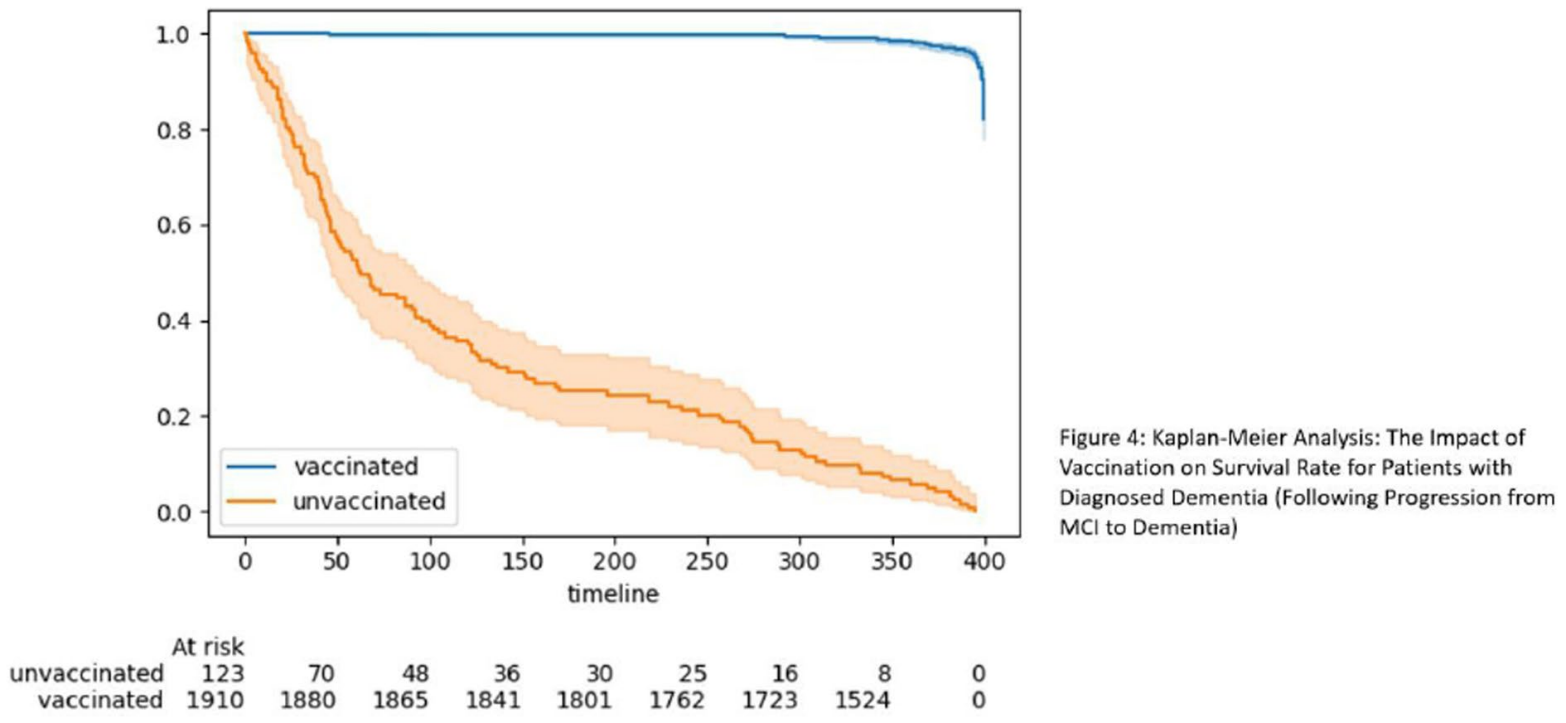
Kaplan–Meier analysis: the impact of vaccination on survival rate for patients with diagnosed dementia (following progression from MCI to dementia).

The aforementioned findings explain the data shown in Tables 1, 2. Namely, despite a higher proportion of vaccinated individuals testing positive for COVID-19, their survival rate was still higher. Our final investigation centered on individuals diagnosed with dementia, specifically those who transitioned from MCI to dementia (Figure 4). Once again, the results underscored the significance of vaccination, revealing that vaccinated individuals with diagnosed dementia demonstrated a higher survival rate over time. This consistent trend suggests that vaccination continued to play a vital role in enhancing the survival prospects of adults with cognitive disorders during the COVID-19 pandemic.

## Discussion

The study aimed to assess the impact of COVID-19 vaccination on older adults with cognitive disorders by examining various health-related variables and outcomes. During the pandemic, older adults with dementia, including those with Alzheimer’s disease and vascular dementia were at increased risk for COVID-19 infection and severe outcomes (23), even after controlling for demographic factors and comorbidities (17). Our results align with previous research and provide compelling evidence that COVID-19 vaccination is significantly associated with a reduced risk of mortality in individuals with cognitive disorders (16). Furthermore, the results indicate a plausible connection between vaccination and safeguarding against a subsequent deterioration in cognitive disorders and associated comorbidities. However, this effect was not evident when examining the transition from MCI to dementia.

Consistent with previous research, our study observed higher COVID-19 positivity rates among vaccinated individuals (“breakthrough infections”) (17). These could be due to several reasons. For example, vaccinated individuals may have lower hesitancy engaging in social activities/travel, leading to higher exposure risk. Additionally, imperfect protection from vaccines could allow more breakthrough infections to occur. Waning immunity over time may also leave some vaccinated individuals vulnerable, supporting the need for boosters. Variants with greater vaccine evasion properties, such as Omicron, could disproportionately infect vaccinated persons as well (17, 24).

However, despite the higher incidence of infection, vaccinated individuals demonstrated significantly lower mortality rates compared to their unvaccinated counterparts. These results further support the notion that vaccination may confer a protective effect against severe outcomes and reduce the risk of mortality (16). Moreover, the results suggest that the increased risk of breakthrough infections in older adults with dementia, compared to those without dementia, can be mainly attributed to their higher prevalence of comorbidities (17, 25). Studies have found an association between COVID-19 vaccination and reduced risk of severe outcomes in people with comorbidities like diabetes and hypertension (26).

Understanding the significance of these underlying health conditions is crucial in comprehending the susceptibility of individuals with dementia to COVID-19 breakthrough infections (27). Addressing these comorbidities and implementing appropriate preventive measures may be vital in mitigating the risk of infection and its potential complications in this vulnerable population. Furthermore, vaccination appeared to impact the management of certain health conditions. In particular, vaccinated individuals with cognitive disorders demonstrated a lower percentage of diabetes cases compared to their unvaccinated counterparts. This observation aligns with studies suggesting that COVID-19 vaccination may have additional health benefits beyond its direct impact on COVID-19 infection (18).

The potential benefits of mental health support observed in our study are intriguing and warrant further investigation. Vaccinated individuals displayed differences in antipsychotics drugs usage and prescription rates compared to the unvaccinated group, suggesting a possible association between vaccination and improved mental health outcomes (28–30). Although the underlying mechanisms are not fully understood, this finding corroborates with previous studies that have explored the broader impact of vaccination on mental health during the pandemic (18, 31).

The results of this study are important for several reasons. First, they provide further evidence that vaccination is an effective way to protect against severe outcomes of COVID-19, even in vulnerable populations such as those with cognitive disorders. Second, the results suggest that vaccination may also have benefits for cognitive health, although further research is needed to confirm this finding. For example, population-based matched cohort studies with longer follow-up periods can better establish the long-term effects of COVID-19 vaccines on mortality and critical disease endpoints in people with dementia.

Finally, the results of this study highlight the importance of vaccination for adults with cognitive disorders who are at increased risk of severe illness and death from COVID-19.

### Strengths

The results of this study highlight the importance of monitoring cognitive health in adults with cognitive disorders who are vaccinated. While vaccination may help reduce the risks of severe COVID-19 infection and related mortality in this population, there is some evidence it may also be associated with a higher risk of certain psychiatric adverse events, like depression and anxiety (32).

However, the potential increased psychiatric risks should be balanced against the benefits of reduced mortality and severe disease afforded by vaccination for this vulnerable group. Healthcare providers need to be cognizant of the possibility of new or worsening mental health symptoms following vaccination among patients with cognitive disorders. Screening for changes in mental status could enable early intervention. More research is still needed to better understand the mental health impacts of COVID-19 vaccination across populations. Additionally, analyses of the effects of vaccination on all-cause mortality rates in adults with cognitive disorders compared to the general population may provide further insight into the risk–benefit ratio and help inform vaccination policies for this group.

### Limitations

It is important to note that the results of this study are observational, and therefore cannot be used to establish causal relationships. However, the consistency of the findings across both the research and control groups, as well as the support from the Kaplan–Meier analyses, suggest that vaccination is likely to be an important factor in the observed lower mortality rates. Second, we were unable to distinguish the effect of vaccination by specific SARS-CoV-2 variant due to a lack of data on the viral strains participants were infected with. Furthermore, we did not have access to patient medical records detailing discharge diagnoses and primary reasons for hospital admission. As such, we were unable to distinguish whether observed differences in hospitalization rates between the vaccinated and unvaccinated groups were specifically due to SARS-CoV-2 infection or other underlying medical conditions.

Finally, vaccination status could potentially confound the association between COVID-19 infection and quality of life outcomes. Vaccinated patients are more likely to have fewer restrictions imposed on them and greater freedom to participate in activities that may improve quality of life (33, 34). Therefore, some of the observed differences in quality of life between infected and uninfected groups could be due to baseline differences in vaccination status, rather than due solely to COVID-19 infection effects. Future studies should measure and control for vaccination status when examining the impacts of COVID-19 on quality of life outcomes.

## Conclusion

This study provides strong evidence that COVID-19 vaccination is beneficial for individuals with cognitive disorders. Although vaccinated individuals had higher COVID-19 positivity rates, they had significantly lower mortality rates, highlighting the vaccine’s protective effect against severe outcomes. These findings are consistent with recent research on the vaccine’s efficacy in preventing severe illness and death. Additionally, the study suggests a potential association between vaccination and a lower risk of mental health conditions, although further investigation is needed. Addressing underlying health conditions and implementing preventive measures are crucial in reducing breakthrough infections in older adults with dementia. Overall, this study emphasizes the vital role of vaccination in protecting vulnerable populations, such as those with cognitive disorders, during the COVID-19 pandemic.

## Data availability statement

The raw data supporting the conclusions of this article will be made available by the authors, without undue reservation.

## Ethics statement

The study protocol was approved by the Institutional Human Subjects Ethics Committee (0075-22-MHS) of the relevant medical facility. The studies were conducted in accordance with the local legislation and institutional requirements. Written informed consent for participation was not required from the participants or the participants’ legal guardians/next of kin in accordance with the national legislation and institutional requirements.

## Author contributions

ZR: Conceptualization, Investigation, Methodology, Project administration, Supervision, Writing – review & editing. SK: Project administration, Supervision, Writing – review & editing. SL: Conceptualization, Writing – review & editing. NB: Conceptualization, Writing – review & editing. LK: Conceptualization, Writing – review & editing. RN-G: Conceptualization, Investigation, Writing – review & editing. SS: Conceptualization, Investigation, Writing – review & editing. IH: Conceptualization, Investigation, Writing – review & editing. OR: Investigation, Supervision, Writing – review & editing. YA: Formal analysis, Methodology, Writing – original draft. MS: Conceptualization, Methodology, Validation, Writing – original draft.
