# Supplementary material for: Association between COVID-19 vaccination and critical outcomes among older adults with dementia: a comparative cohort study
Source: Front Public Health. 2023 Oct 2;11:1281266. doi: 10.3389/fpubh.2023.1281266 (PMC10578450; doi:10.3389/fpubh.2023.1281266)
Supplement: Supplementary file 1 [file Table_1.DOCX]

**Appendix 1. Dementia scoring tool.**

**Entrance criteria:**

• Age above 45

• Diagnoses of dementia according to disease type/MCI

- High certainty
- Active diagnosis or two diagnostic visits:
- Geriatrician, psychiatrist, neurologist.
- Family physician with a suitable SWEET 16 score (below 13)
- Diagnosis from medication approvals
- Low certainty
- Active diagnosis or two diagnostic visits from a family physician without test results

• Medications- Purchase of medication from the group of anti-dementia drugs

Follow-ups:

- Association with other records - heart, diabetes, pediatric, bone disease, and more.
- Company and billing notes - discretion, complex patient, foreign worker, nursing home, assisted living, care act.
- Medications - anti-dementia, antipsychotic, anti-anxiety/antidepressant.
- Laboratories - complete blood count, hemoglobin, iron, B12, TSH, albumin, and more.
- Visits - family physician, geriatrician, neurologist, psychiatrist, dietitian, and more.
- BMI.
- ADL.
- Depression.
- Cognitive tests - SW16, MMSE.
